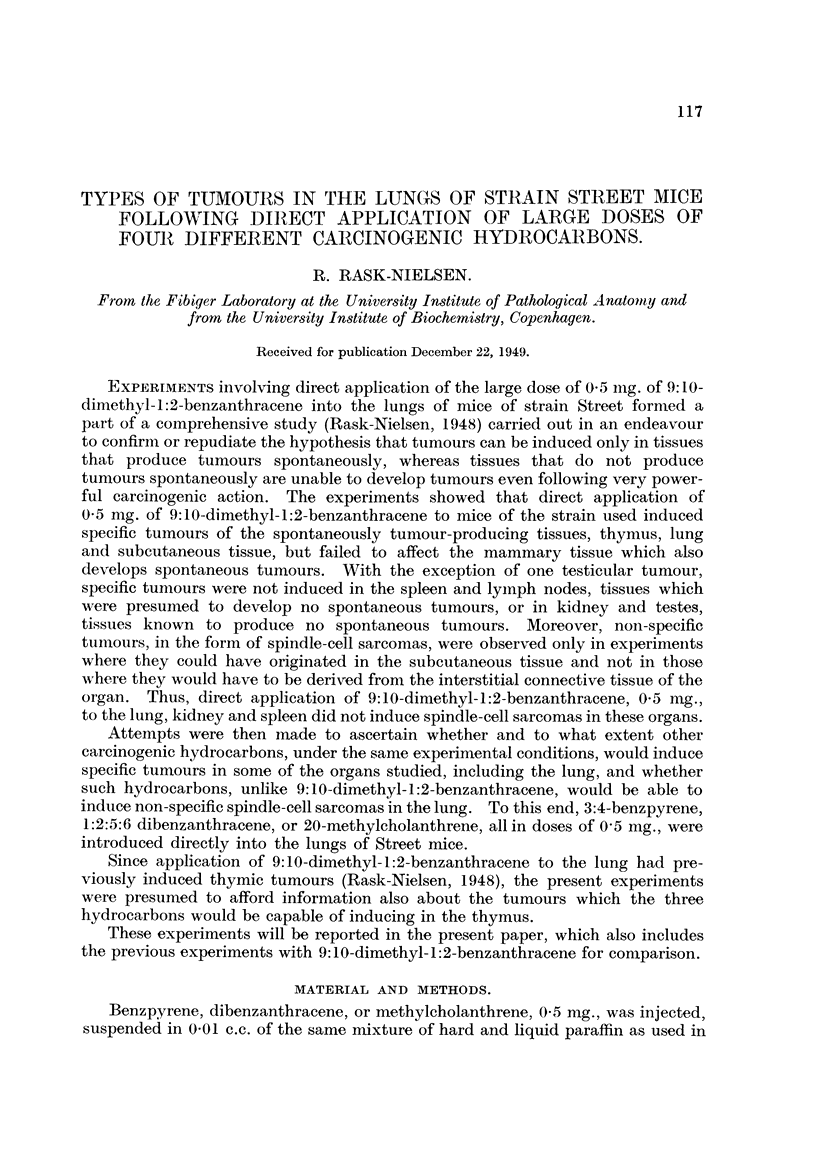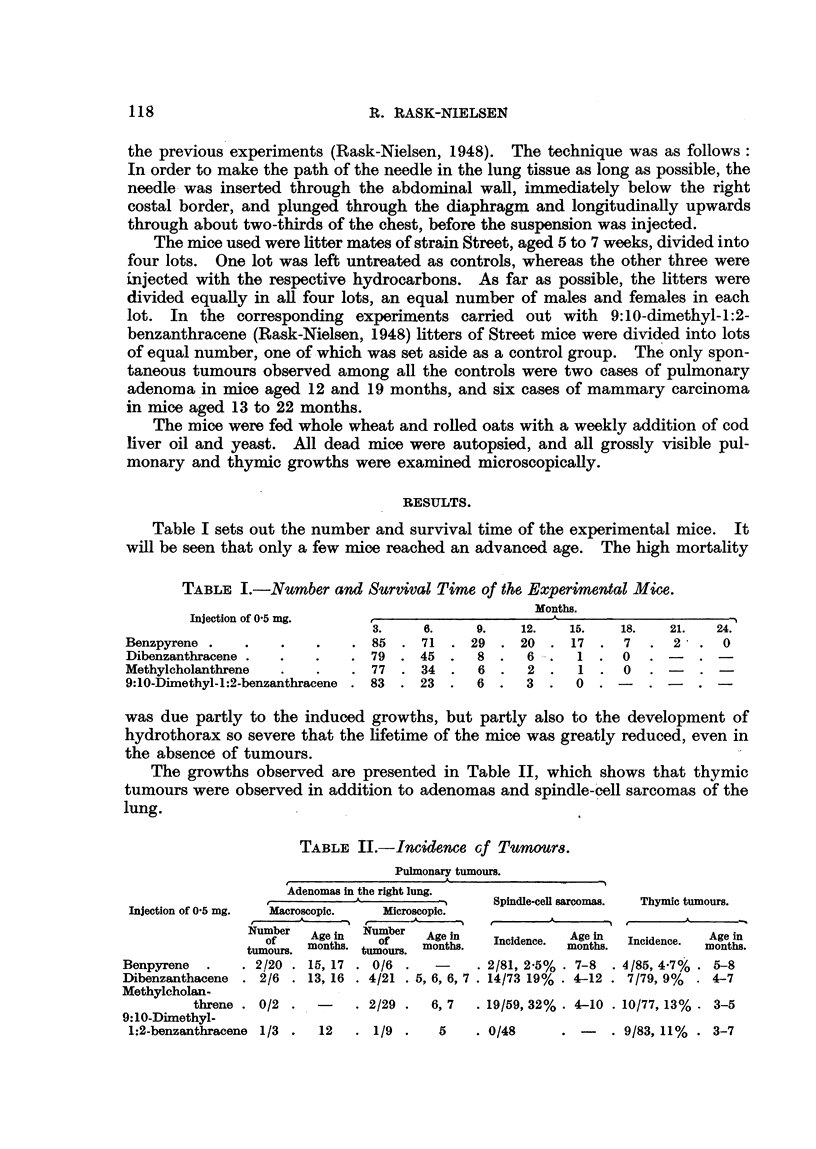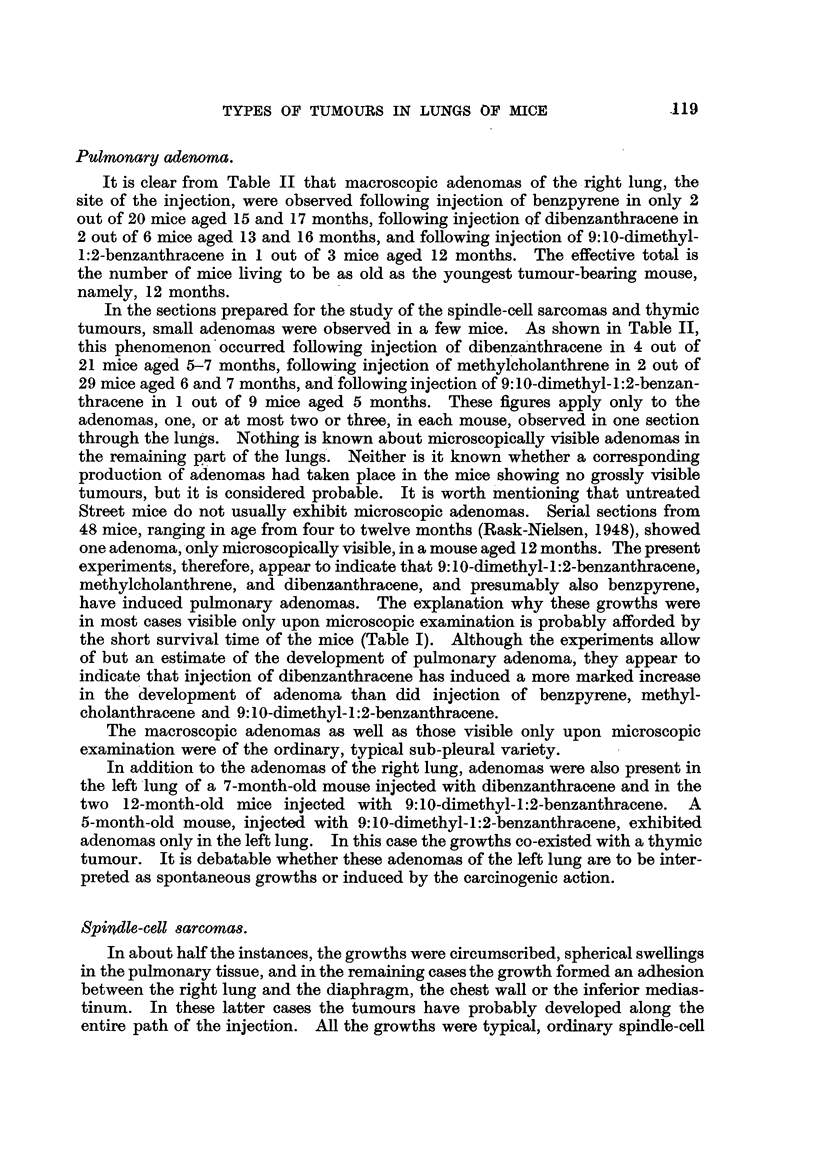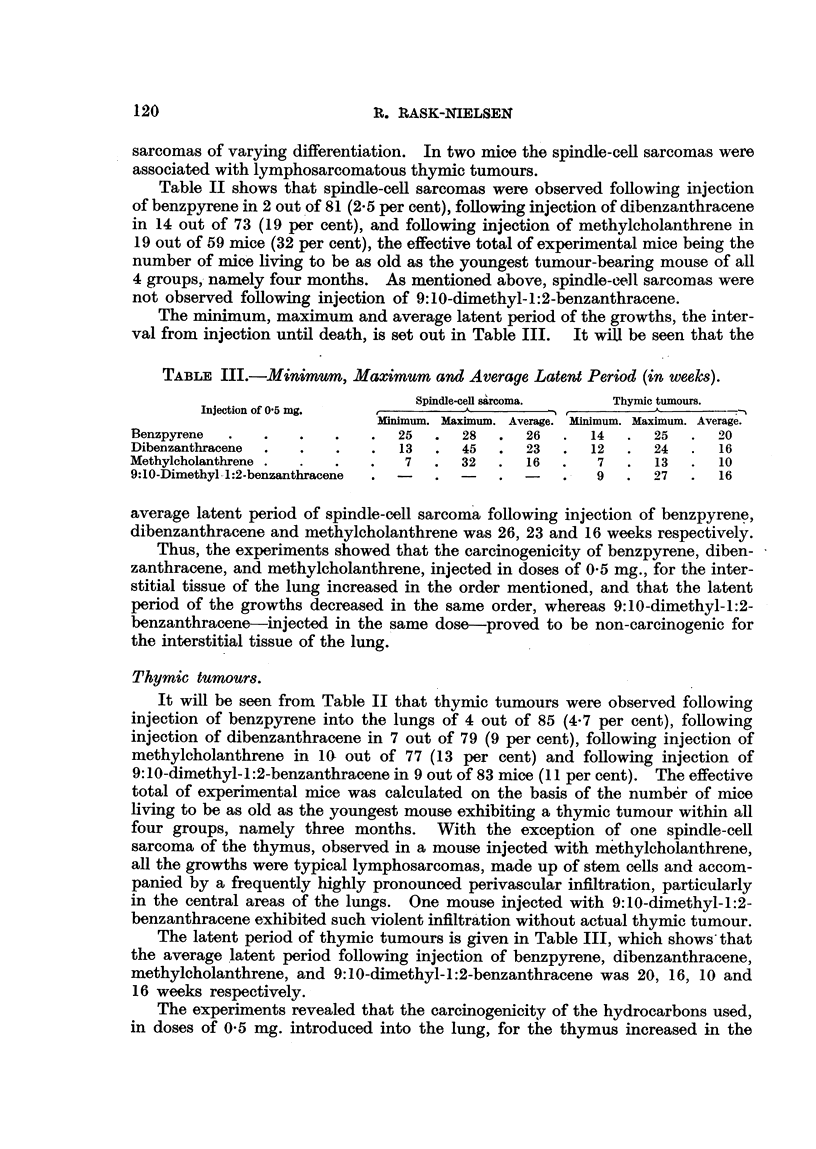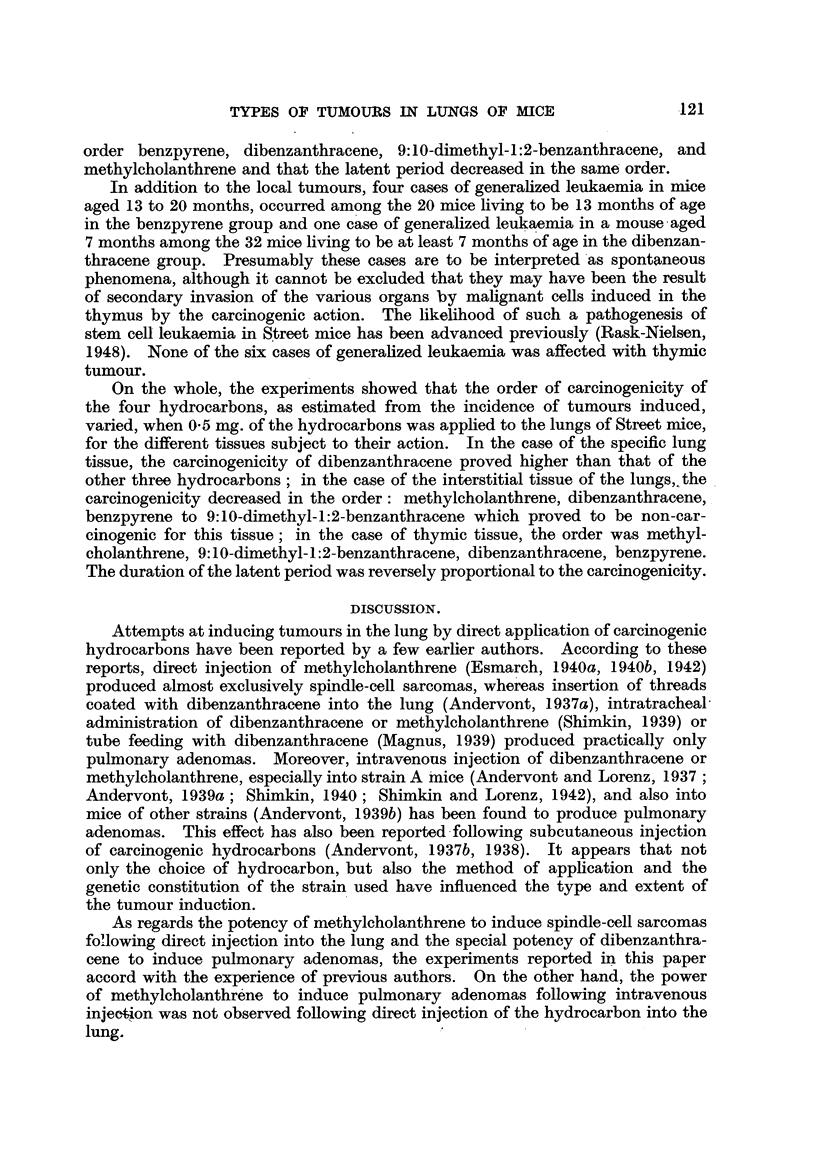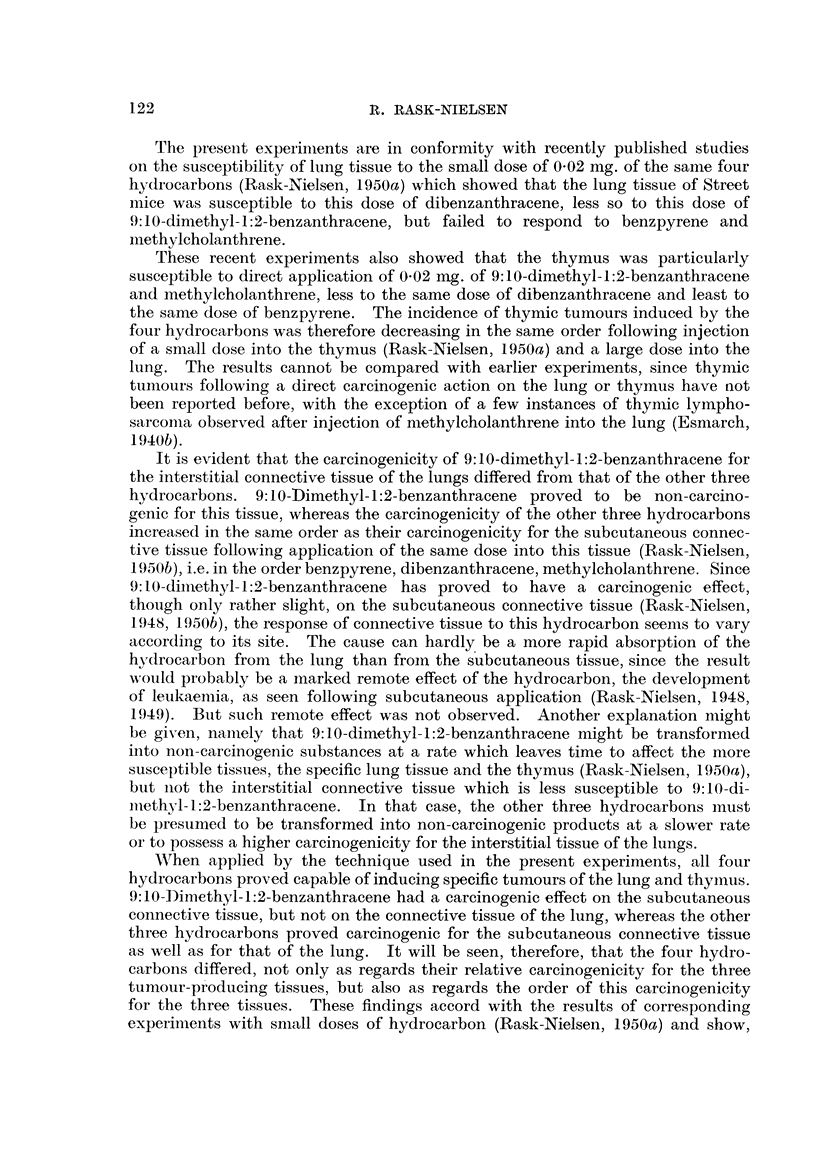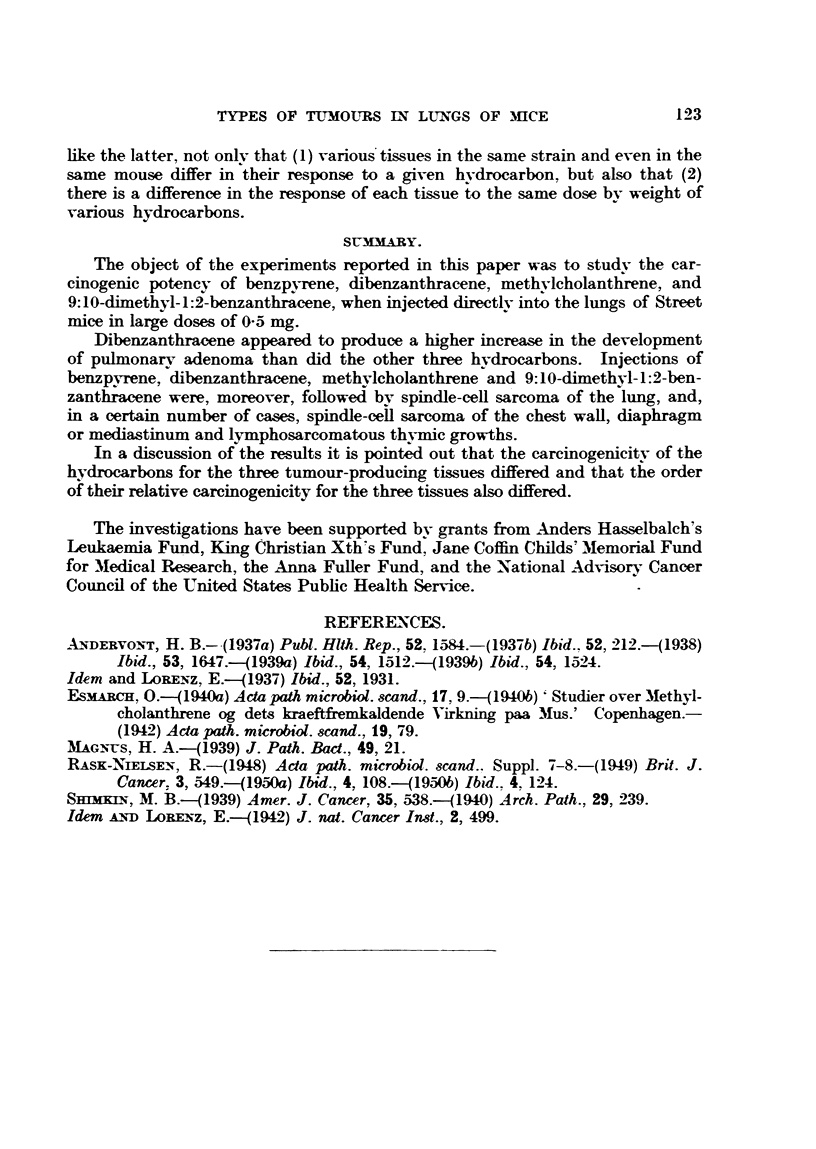# Types of Tumours in the Lungs of Strain Street Mice following Direct Application of Large Doses of Four Different Carcinogenic Hydrocarbons

**DOI:** 10.1038/bjc.1950.11

**Published:** 1950-03

**Authors:** R. Rask-Nielsen


### TYPES OF TUMOURS IN THE LUNGS OF STRAIN STREET MICE FOLLOWING DIRECT APPLICATION OF LARGE DOSES OF FOUR DIFFERENT CARCINOGENIC HYDROCARBONS.

R. RASK-NIELSEN.

*From the Fibiger Laboratory at the University Institute of Pathological Anatomy and from the University Institute of Biochemistry, Copenhagen.*

Received for publication December 22, 1949.

EXPERIMENTS involving direct application of the large dose of 0·5 mg. of 9:10-dimethyl-1:2-benzanthracene into the lungs of mice of strain Street formed a part of a comprehensive study (Rask-Nielsen, 1948) carried out in an endeavour to confirm or repudiate the hypothesis that tumours can be induced only in tissues that produce tumours spontaneously, whereas tissues that do not produce tumours spontaneously are unable to develop tumours even following very powerful carcinogenic action. The experiments showed that direct application of 0·5 mg. of 9:10-dimethyl-1:2-benzanthracene to mice of the strain used induced specific tumours of the spontaneously tumour-producing tissues, thymus, lung and subcutaneous tissue, but failed to affect the mammary tissue which also develops spontaneous tumours. With the exception of one testicular tumour, specific tumours were not induced in the spleen and lymph nodes, tissues which were presumed to develop no spontaneous tumours, or in kidney and testes, tissues known to produce no spontaneous tumours. Moreover, non-specific tumours, in the form of spindle-cell sarcomas, were observed only in experiments where they could have originated in the subcutaneous tissue and not in those where they would have to be derived from the interstitial connective tissue of the organ. Thus, direct application of 9:10-dimethyl-1:2-benzanthracene, 0·5 mg., to the lung, kidney and spleen did not induce spindle-cell sarcomas in these organs.

Attempts were then made to ascertain whether and to what extent other carcinogenic hydrocarbons, under the same experimental conditions, would induce specific tumours in some of the organs studied, including the lung, and whether such hydrocarbons, unlike 9:10-dimethyl-1:2-benzanthracene, would be able to induce non-specific spindle-cell sarcomas in the lung. To this end, 3:4-benzpyrene, 1:2:5:6 dibenzanthracene, or 20-methylcholanthrene, all in doses of 0·5 mg., were introduced directly into the lungs of Street mice.

Since application of 9:10-dimethyl-1:2-benzanthracene to the lung had previously induced thymic tumours (Rask-Nielsen, 1948), the present experiments were presumed to afford information also about the tumours which the three hydrocarbons would be capable of inducing in the thymus.

These experiments will be reported in the present paper, which also includes the previous experiments with 9:10-dimethyl-1:2-benzanthracene for comparison.

### MATERIAL AND METHODS.

Benzpyrene, dibenzanthracene, or methylcholanthrene, 0·5 mg., was injected, suspended in 0·01 c.c. of the same mixture of hard and liquid paraffin as used in the previous experiments (Rask-Nielsen, 1948). The technique was as follows : In order to make the path of the needle in the lung tissue as long as possible, the needle was inserted through the abdominal wall, immediately below the right costal border, and plunged through the diaphragm and longitudinally upwards through about two-thirds of the chest, before the suspension was injected.

The mice used were litter mates of strain Street, aged 5 to 7 weeks, divided into four lots. One lot was left untreated as controls, whereas the other three were injected with the respective hydrocarbons. As far as possible, the litters were divided equally in all four lots, an equal number of males and females in each lot. In the corresponding experiments carried out with 9:10-dimethyl-1:2-benzanthracene (Rask-Nielsen, 1948) litters of Street mice were divided into lots of equal number, one of which was set aside as a control group. The only spontaneous tumours observed among all the controls were two cases of pulmonary adenoma in mice aged 12 and 19 months, and six cases of mammary carcinoma in mice aged 13 to 22 months.

The mice were fed whole wheat and rolled oats with a weekly addition of cod liver oil and yeast. All dead mice were autopsied, and all grossly visible pulmonary and thymic growths were examined microscopically.

### RESULTS.

Table I sets out the number and survival time of the experimental mice. It will be seen that only a few mice reached an advanced age. The high mortality

TABLE I.—*Number and Survival Time of the Experimental Mice.*

| Injection of 0·5 mg. | Months. 3. | 6. | 9. | 12. | 15. | 18. | 21. | 24. |
|---|---|---|---|---|---|---|---|---|
| Benzpyrene | 85 | 71 | 29 | 20 | 17 | 7 | 2 | 0 |
| Dibenzanthracene | 79 | 45 | 8 | 6 | 1 | 0 | — | — |
| Methylcholanthrene | 77 | 34 | 6 | 2 | 1 | 0 | — | — |
| 9:10-Dimethyl-1:2-benzanthracene | 83 | 23 | 6 | 3 | 0 | — | — | — |

was due partly to the induced growths, but partly also to the development of hydrothorax so severe that the lifetime of the mice was greatly reduced, even in the absence of tumours.

The growths observed are presented in Table II, which shows that thymic tumours were observed in addition to adenomas and spindle-cell sarcomas of the lung.

TABLE II.—*Incidence cf Tumours.*

| Injection of 0·5 mg. | Pulmonary tumours. Adenomas in the right lung. Macroscopic. Number of tumours. | Age in months. | Microscopic. Number of tumours. | Age in months. | Spindle-cell sarcomas. Incidence. | Age in months. | Thymic tumours. Incidence. | Age in months. |
|---|---|---|---|---|---|---|---|---|
| Benpyrene | 2/20 | 15, 17 | 0/6 | — | 2/81, 2·5% | 7–8 | 4/85, 4·7% | 5–8 |
| Dibenzanthacene | 2/6 | 13, 16 | 4/21 | 5, 6, 6, 7 | 14/73 19% | 4–12 | 7/79, 9% | 4–7 |
| Methylcholanthrene | 0/2 | — | 2/29 | 6, 7 | 19/59, 32% | 4–10 | 10/77, 13% | 3–5 |
| 9:10-Dimethyl-1:2-benzanthracene | 1/3 | 12 | 1/9 | 5 | 0/48 | — | 9/83, 11% | 3–7 |

*Pulmonary adenoma.*

It is clear from Table II that macroscopic adenomas of the right lung, the site of the injection, were observed following injection of benzpyrene in only 2 out of 20 mice aged 15 and 17 months, following injection of dibenzanthracene in 2 out of 6 mice aged 13 and 16 months, and following injection of 9:10-dimethyl-1:2-benzanthracene in 1 out of 3 mice aged 12 months. The effective total is the number of mice living to be as old as the youngest tumour-bearing mouse, namely, 12 months.

In the sections prepared for the study of the spindle-cell sarcomas and thymic tumours, small adenomas were observed in a few mice. As shown in Table II, this phenomenon occurred following injection of dibenzanthracene in 4 out of 21 mice aged 5–7 months, following injection of methylcholanthrene in 2 out of 29 mice aged 6 and 7 months, and following injection of 9:10-dimethyl-1:2-benzanthracene in 1 out of 9 mice aged 5 months. These figures apply only to the adenomas, one, or at most two or three, in each mouse, observed in one section through the lungs. Nothing is known about microscopically visible adenomas in the remaining part of the lungs. Neither is it known whether a corresponding production of adenomas had taken place in the mice showing no grossly visible tumours, but it is considered probable. It is worth mentioning that untreated Street mice do not usually exhibit microscopic adenomas. Serial sections from 48 mice, ranging in age from four to twelve months (Rask-Nielsen, 1948), showed one adenoma, only microscopically visible, in a mouse aged 12 months. The present experiments, therefore, appear to indicate that 9:10-dimethyl-1:2-benzanthracene, methylcholanthrene, and dibenzanthracene, and presumably also benzpyrene, have induced pulmonary adenomas. The explanation why these growths were in most cases visible only upon microscopic examination is probably afforded by the short survival time of the mice (Table I). Although the experiments allow of but an estimate of the development of pulmonary adenoma, they appear to indicate that injection of dibenzanthracene has induced a more marked increase in the development of adenoma than did injection of benzpyrene, methylcholanthracene and 9:10-dimethyl-1:2-benzanthracene.

The macroscopic adenomas as well as those visible only upon microscopic examination were of the ordinary, typical sub-pleural variety.

In addition to the adenomas of the right lung, adenomas were also present in the left lung of a 7-month-old mouse injected with dibenzanthracene and in the two 12-month-old mice injected with 9:10-dimethyl-1:2-benzanthracene. A 5-month-old mouse, injected with 9:10-dimethyl-1:2-benzanthracene, exhibited adenomas only in the left lung. In this case the growths co-existed with a thymic tumour. It is debatable whether these adenomas of the left lung are to be interpreted as spontaneous growths or induced by the carcinogenic action.

*Spindle-cell sarcomas.*

In about half the instances, the growths were circumscribed, spherical swellings in the pulmonary tissue, and in the remaining cases the growth formed an adhesion between the right lung and the diaphragm, the chest wall or the inferior mediastinum. In these latter cases the tumours have probably developed along the entire path of the injection. All the growths were typical, ordinary spindle-cell sarcomas of varying differentiation. In two mice the spindle-cell sarcomas were associated with lymphosarcomatous thymic tumours.

Table II shows that spindle-cell sarcomas were observed following injection of benzpyrene in 2 out of 81 (2·5 per cent), following injection of dibenzanthracene in 14 out of 73 (19 per cent), and following injection of methylcholanthrene in 19 out of 59 mice (32 per cent), the effective total of experimental mice being the number of mice living to be as old as the youngest tumour-bearing mouse of all 4 groups, namely four months. As mentioned above, spindle-cell sarcomas were not observed following injection of 9:10-dimethyl-1:2-benzanthracene.

The minimum, maximum and average latent period of the growths, the interval from injection until death, is set out in Table III. It will be seen that the

TABLE III.—*Minimum, Maximum and Average Latent Period (in weeks).*

| Injection of 0·5 mg. | Spindle-cell sarcoma. | | | Thymic tumours. | | |
|---|---|---|---|---|---|---|
| | Minimum. | Maximum. | Average. | Minimum. | Maximum. | Average. |
| Benzpyrene | 25 | 28 | 26 | 14 | 25 | 20 |
| Dibenzanthracene | 13 | 45 | 23 | 12 | 24 | 16 |
| Methylcholanthrene | 7 | 32 | 16 | 7 | 13 | 10 |
| 9:10-Dimethyl-1:2-benzanthracene | — | — | — | 9 | 27 | 16 |

average latent period of spindle-cell sarcoma following injection of benzpyrene, dibenzanthracene and methylcholanthrene was 26, 23 and 16 weeks respectively.

Thus, the experiments showed that the carcinogenicity of benzpyrene, dibenzanthracene, and methylcholanthrene, injected in doses of 0·5 mg., for the interstitial tissue of the lung increased in the order mentioned, and that the latent period of the growths decreased in the same order, whereas 9:10-dimethyl-1:2-benzanthracene—injected in the same dose—proved to be non-carcinogenic for the interstitial tissue of the lung.

*Thymic tumours.*

It will be seen from Table II that thymic tumours were observed following injection of benzpyrene into the lungs of 4 out of 85 (4·7 per cent), following injection of dibenzanthracene in 7 out of 79 (9 per cent), following injection of methylcholanthrene in 10 out of 77 (13 per cent) and following injection of 9:10-dimethyl-1:2-benzanthracene in 9 out of 83 mice (11 per cent). The effective total of experimental mice was calculated on the basis of the number of mice living to be as old as the youngest mouse exhibiting a thymic tumour within all four groups, namely three months. With the exception of one spindle-cell sarcoma of the thymus, observed in a mouse injected with methylcholanthrene, all the growths were typical lymphosarcomas, made up of stem cells and accompanied by a frequently highly pronounced perivascular infiltration, particularly in the central areas of the lungs. One mouse injected with 9:10-dimethyl-1:2-benzanthracene exhibited such violent infiltration without actual thymic tumour.

The latent period of thymic tumours is given in Table III, which shows that the average latent period following injection of benzpyrene, dibenzanthracene, methylcholanthrene, and 9:10-dimethyl-1:2-benzanthracene was 20, 16, 10 and 16 weeks respectively.

The experiments revealed that the carcinogenicity of the hydrocarbons used, in doses of 0·5 mg. introduced into the lung, for the thymus increased in the order benzpyrene, dibenzanthracene, 9:10-dimethyl-1:2-benzanthracene, and methylcholanthrene and that the latent period decreased in the same order.

In addition to the local tumours, four cases of generalized leukaemia in mice aged 13 to 20 months, occurred among the 20 mice living to be 13 months of age in the benzpyrene group and one case of generalized leukaemia in a mouse aged 7 months among the 32 mice living to be at least 7 months of age in the dibenzanthracene group. Presumably these cases are to be interpreted as spontaneous phenomena, although it cannot be excluded that they may have been the result of secondary invasion of the various organs by malignant cells induced in the thymus by the carcinogenic action. The likelihood of such a pathogenesis of stem cell leukaemia in Street mice has been advanced previously (Rask-Nielsen, 1948). None of the six cases of generalized leukaemia was affected with thymic tumour.

On the whole, the experiments showed that the order of carcinogenicity of the four hydrocarbons, as estimated from the incidence of tumours induced, varied, when 0·5 mg. of the hydrocarbons was applied to the lungs of Street mice, for the different tissues subject to their action. In the case of the specific lung tissue, the carcinogenicity of dibenzanthracene proved higher than that of the other three hydrocarbons ; in the case of the interstitial tissue of the lungs, the carcinogenicity decreased in the order : methylcholanthrene, dibenzanthracene, benzpyrene to 9:10-dimethyl-1:2-benzanthracene which proved to be non-carcinogenic for this tissue ; in the case of thymic tissue, the order was methylcholanthrene, 9:10-dimethyl-1:2-benzanthracene, dibenzanthracene, benzpyrene. The duration of the latent period was reversely proportional to the carcinogenicity.

### DISCUSSION.

Attempts at inducing tumours in the lung by direct application of carcinogenic hydrocarbons have been reported by a few earlier authors. According to these reports, direct injection of methylcholanthrene (Esmarch, 1940*a*, 1940*b*, 1942) produced almost exclusively spindle-cell sarcomas, whereas insertion of threads coated with dibenzanthracene into the lung (Andervont, 1937*a*), intratracheal administration of dibenzanthracene or methylcholanthrene (Shimkin, 1939) or tube feeding with dibenzanthracene (Magnus, 1939) produced practically only pulmonary adenomas. Moreover, intravenous injection of dibenzanthracene or methylcholanthrene, especially into strain A mice (Andervont and Lorenz, 1937 ; Andervont, 1939*a* ; Shimkin, 1940 ; Shimkin and Lorenz, 1942), and also into mice of other strains (Andervont, 1939*b*) has been found to produce pulmonary adenomas. This effect has also been reported following subcutaneous injection of carcinogenic hydrocarbons (Andervont, 1937*b*, 1938). It appears that not only the choice of hydrocarbon, but also the method of application and the genetic constitution of the strain used have influenced the type and extent of the tumour induction.

As regards the potency of methylcholanthrene to induce spindle-cell sarcomas following direct injection into the lung and the special potency of dibenzanthracene to induce pulmonary adenomas, the experiments reported in this paper accord with the experience of previous authors. On the other hand, the power of methylcholanthrene to induce pulmonary adenomas following intravenous injection was not observed following direct injection of the hydrocarbon into the lung.

The present experiments are in conformity with recently published studies on the susceptibility of lung tissue to the small dose of 0·02 mg. of the same four hydrocarbons (Rask-Nielsen, 1950*a*) which showed that the lung tissue of Street mice was susceptible to this dose of dibenzanthracene, less so to this dose of 9:10-dimethyl-1:2-benzanthracene, but failed to respond to benzpyrene and methylcholanthrene.

These recent experiments also showed that the thymus was particularly susceptible to direct application of 0·02 mg. of 9:10-dimethyl-1:2-benzanthracene and methylcholanthrene, less to the same dose of dibenzanthracene and least to the same dose of benzpyrene. The incidence of thymic tumours induced by the four hydrocarbons was therefore decreasing in the same order following injection of a small dose into the thymus (Rask-Nielsen, 1950*a*) and a large dose into the lung. The results cannot be compared with earlier experiments, since thymic tumours following a direct carcinogenic action on the lung or thymus have not been reported before, with the exception of a few instances of thymic lymphosarcoma observed after injection of methylcholanthrene into the lung (Esmarch, 1940*b*).

It is evident that the carcinogenicity of 9:10-dimethyl-1:2-benzanthracene for the interstitial connective tissue of the lungs differed from that of the other three hydrocarbons. 9:10-Dimethyl-1:2-benzanthracene proved to be non-carcinogenic for this tissue, whereas the carcinogenicity of the other three hydrocarbons increased in the same order as their carcinogenicity for the subcutaneous connective tissue following application of the same dose into this tissue (Rask-Nielsen, 1950*b*), i.e. in the order benzpyrene, dibenzanthracene, methylcholanthrene. Since 9:10-dimethyl-1:2-benzanthracene has proved to have a carcinogenic effect, though only rather slight, on the subcutaneous connective tissue (Rask-Nielsen, 1948, 1950*b*), the response of connective tissue to this hydrocarbon seems to vary according to its site. The cause can hardly be a more rapid absorption of the hydrocarbon from the lung than from the subcutaneous tissue, since the result would probably be a marked remote effect of the hydrocarbon, the development of leukaemia, as seen following subcutaneous application (Rask-Nielsen, 1948, 1949). But such remote effect was not observed. Another explanation might be given, namely that 9:10-dimethyl-1:2-benzanthracene might be transformed into non-carcinogenic substances at a rate which leaves time to affect the more susceptible tissues, the specific lung tissue and the thymus (Rask-Nielsen, 1950*a*), but not the interstitial connective tissue which is less susceptible to 9:10-dimethyl-1:2-benzanthracene. In that case, the other three hydrocarbons must be presumed to be transformed into non-carcinogenic products at a slower rate or to possess a higher carcinogenicity for the interstitial tissue of the lungs.

When applied by the technique used in the present experiments, all four hydrocarbons proved capable of inducing specific tumours of the lung and thymus. 9:10-Dimethyl-1:2-benzanthracene had a carcinogenic effect on the subcutaneous connective tissue, but not on the connective tissue of the lung, whereas the other three hydrocarbons proved carcinogenic for the subcutaneous connective tissue as well as for that of the lung. It will be seen, therefore, that the four hydrocarbons differed, not only as regards their relative carcinogenicity for the three tumour-producing tissues, but also as regards the order of this carcinogenicity for the three tissues. These findings accord with the results of corresponding experiments with small doses of hydrocarbon (Rask-Nielsen, 1950*a*) and show, like the latter, not only that (1) various tissues in the same strain and even in the same mouse differ in their response to a given hydrocarbon, but also that (2) there is a difference in the response of each tissue to the same dose by weight of various hydrocarbons.

### SUMMARY.

The object of the experiments reported in this paper was to study the carcinogenic potency of benzpyrene, dibenzanthracene, methylcholanthrene, and 9:10-dimethyl-1:2-benzanthracene, when injected directly into the lungs of Street mice in large doses of 0·5 mg.

Dibenzanthracene appeared to produce a higher increase in the development of pulmonary adenoma than did the other three hydrocarbons. Injections of benzpyrene, dibenzanthracene, methylcholanthrene and 9:10-dimethyl-1:2-benzanthracene were, moreover, followed by spindle-cell sarcoma of the lung, and, in a certain number of cases, spindle-cell sarcoma of the chest wall, diaphragm or mediastinum and lymphosarcomatous thymic growths.

In a discussion of the results it is pointed out that the carcinogenicity of the hydrocarbons for the three tumour-producing tissues differed and that the order of their relative carcinogenicity for the three tissues also differed.

The investigations have been supported by grants from Anders Hasselbalch's Leukaemia Fund, King Christian Xth's Fund, Jane Coffin Childs' Memorial Fund for Medical Research, the Anna Fuller Fund, and the National Advisory Cancer Council of the United States Public Health Service.

### REFERENCES.

ANDERVONT, H. B.—(1937*a*) *Publ. Hlth. Rep.*, **52**, 1584.—(1937*b*) *Ibid.*, **52**, 212.—(1938) *Ibid.*, **53**, 1647.—(1939*a*) *Ibid.*, **54**, 1512.—(1939*b*) *Ibid.*, **54**, 1524.

*Idem* and LORENZ, E.—(1937) *Ibid.*, **52**, 1931.

ESMARCH, O.—(1940*a*) *Acta path microbiol. scand.*, **17**, 9.—(1940*b*) 'Studier over Methylcholanthrene og dets kraeftfremkaldende Virkning paa Mus.' Copenhagen.—(1942) *Acta path. microbiol. scand.*, **19**, 79.

MAGNUS, H. A.—(1939) *J. Path. Bact.*, **49**, 21.

RASK-NIELSEN, R.—(1948) *Acta path. microbiol. scand.*, Suppl. 7–8.—(1949) *Brit. J. Cancer*, **3**, 549.—(1950*a*) *Ibid.*, **4**, 108.—(1950*b*) *Ibid.*, **4**, 124.

SHIMKIN, M. B.—(1939) *Amer. J. Cancer*, **35**, 538.—(1940) *Arch. Path.*, **29**, 239.

*Idem* AND LORENZ, E.—(1942) *J. nat. Cancer Inst.*, **2**, 499.